# Developing an Unguided Internet-Delivered Intervention for Emotional Distress in Primary Care Patients: Applying Common Factor and Person-Based Approaches

**DOI:** 10.2196/mental.5845

**Published:** 2016-12-20

**Authors:** Adam WA Geraghty, Ricardo F Muñoz, Lucy Yardley, Jennifer Mc Sharry, Paul Little, Michael Moore

**Affiliations:** ^1^ Primary Care and Population Sciences University of Southampton Southampton United Kingdom; ^2^ Institute for International Internet Interventions for Health Palo Alto University Palo Alto, CA United States; ^3^ Department of Psychiatry University of California, San Francisco San Francisco, CA United States; ^4^ Centre for Applications of Health Psychology Academic Unit of Psychology University of Southampton Southampton United Kingdom; ^5^ Health Behaviour Change Research Group NUI Galway Galway Ireland

**Keywords:** Internet, unguided, distress, person-based approach

## Abstract

**Background:**

Developing effective, unguided Internet interventions for mental health represents a challenge. Without structured human guidance, engagement with these interventions is often limited and the effectiveness reduced. If their effectiveness can be increased, they have great potential for broad, low-cost dissemination. Improving unguided Internet interventions for mental health requires a renewed focus on the proposed underlying mechanisms of symptom improvement and the involvement of target users from the outset.

**Objective:**

The aim of our study was to develop an unguided e-mental health intervention for distress in primary care patients, drawing on meta-theory of psychotherapeutic change and utilizing the person-based approach (PBA) to guide iterative qualitative piloting with patients.

**Methods:**

Common factors meta-theory informed the selection and structure of therapeutic content, enabling flexibility whilst retaining the proposed necessary ingredients for effectiveness. A logic model was designed outlining intervention components and proposed mechanisms underlying improvement. The PBA provided a framework for systematically incorporating target-user perspective into the intervention development. Primary care patients (N=20) who had consulted with emotional distress in the last 12 months took part in exploratory qualitative interviews, and a subsample (n=13) undertook think-aloud interviews with a prototype of the intervention.

**Results:**

A flexible intervention was developed, to be used as and when patients need, diverting from a more traditional, linear approach. Based on the in-depth qualitative findings, disorder terms such as “depression” were avoided, and discussions of psychological symptoms were placed in the context of stressful life events. Think-aloud interviews showed that patients were positive about the design and structure of the intervention. On the basis of patient feedback, modifications were made to increase immediate access to all therapeutic techniques.

**Conclusions:**

Detailing theoretical assumptions underlying Internet interventions for mental health, and integrating this approach with systematic in-depth qualitative research with target patients is important. These strategies may provide novel ways for addressing the challenges of unguided delivery. The resulting intervention, Healthy Paths, will be evaluated in primary care-based randomized controlled trials, and deployed as a massive open online intervention (MOOI).

## Introduction

### Background

Research on Internet interventions for common mental health problems is maturing. Numerous systematic reviews have demonstrated the effectiveness of guided Internet interventions for reducing depression and anxiety [1-3], with meta-analyses showing that effect sizes can be equivalent to face-to-face psychotherapy [4]. In contrast, unguided Internet interventions for mental health have been more problematic. Removing structured human guidance frequently results in low use, high attrition, and reduced effectiveness [5-7], leading some to call for unguided formats to be avoided [8]. However, if the challenges of unguided interventions can be addressed, they have the potential to be a low-cost, minimally disruptive, self-directed therapeutic resource [9], with a high chance of successful implementation in health care systems, particularly where resources are limited. This format also has the greatest potential to enable mental health interventions to be delivered globally [10-12]. Addressing the challenges of unguided delivery may require bespoke interventions. The development of such interventions provides opportunities to explore theories underlying therapeutic content and novel development approaches.

The majority of Internet interventions for depression and anxiety emulate face-to-face psychotherapy protocols, predominantly based on cognitive behavior therapy (CBT) [13]. Internet CBT interventions often consist of multiple sessions forming a linear “e-course.” Importantly, the therapeutic content in unguided CBT interventions is usually very similar to their guided counterparts [14]. In an unguided context, without the support and guidance of a therapeutic relationship, critical elements of CBT [15], standard CBT rationales and specific techniques, may be difficult to adhere to [16,17]. This is also likely to be true of other forms of therapy where central ideas are complex (eg, brief psychodynamic therapy) [18]. In order to design effective unguided interventions, the unguided context may need to be considered throughout the development process, with the resulting interventions structured differently and featuring different and/or amended content to guided interventions.

Ideally, when looking to develop a novel unguided Internet intervention, we would turn to the literature on how psychotherapy-derived interventions work; ensuring critical elements are included where possible, and nothing is lost that would have been beneficial. In reality, although there are many evidence-based psychotherapies (EBPs) for depression, [19] there is little evidence regarding which elements of these complex interventions are necessary for effectiveness [20]. Theory may serve as a useful guide. At the level of specific psychotherapeutic approach, however, most EBPs are based on a diverse range of corresponding theories, from Beck’s cognitive theory [21] to Weismann’s interpersonal theory of depression [22], with varying levels of supporting evidence. Consequently, we propose that meta-theory and, in particular, “common factors” models may be helpful [23]. Common factors models arose from psychological, sociological, and anthropological theories around how humans manage disease and reduce distress [24,25]. They comprise sets of key factors and/or ingredients necessary for beneficial change, and have been developed to explain the consistently robust finding that different “active” or bona fide psychotherapeutic interventions lead to equivalent effectiveness [26]. These models represent theories about how psychotherapies in general produce effect; so operate at a higher level of abstraction than the individual theories associated with each therapeutic approach. They may provide a useful overarching guide for amending existing intervention manuals for suitability in an unguided context, whilst retaining their effectiveness.

Although the therapeutic relationship is a key common factor there are a number of remaining factors that transfer well onto unguided Internet interventions including (1) a functional explanation for a person’s symptoms; (2) an aligning coherent therapeutic rationale for the reduction of symptoms; (3) techniques for ameliorating symptoms or training in skills to be utilized to feel more effective in handling daily life; (4) use of techniques and/or skills in the person’s daily life; and (5) attribution of improvement to the person’s increased skillfulness and understanding [24,27,28]. All factors are likely to be important; however, particular emphasis is often placed on the role of explanation and therapeutic rationale in generating positive response expectancies [29-32] and novel insight [33].

Effective interventions such as CBT contain all of the above, although specific theories (eg, cognitive mediation) and practices (eg, correcting automatic thoughts) will be emphasized in treatment protocols. Applying a common factor model enables us to modify, restructure, and potentially remove elements that could be difficult without guidance (automatic thought challenging for instance), whilst ensuring the modified intervention remains within the bounds of a robust meta-theory of effectiveness.

The flexibility inherent in common factor models provides a great deal of scope for the integration of user experience and perspectives when developing unguided Internet interventions. Understanding users’ perceptions of the target symptom as well as the intervention content is likely to be critical for engagement, which is often poor when guidance is removed. The person-based approach (PBA) is a systematic method for integrating in-depth qualitative research into intervention development [34,35]. Emerging from the development of Internet interventions for physical health within health psychology, it aims to ensure interventions are grounded in “a profound understanding of the perspective and psychosocial context of the people who will use them” [35]. The PBA is highly compatible with, and sympathetic to, similar methodologies such as participatory and user-centered design [36,37] in the field of human-computer interaction (HCI). However, the PBA also has significant differences from participatory design methods. The PBA was developed to allow integration of the user perspective into theory- and evidence-based approaches to intervention design. In addition, developing from health psychology, the PBA has an explicit focus on the behavior change and symptom management aspects of user experiences. As such, the PBA can be used in the development of digital and non-digital intervention alike, drawing on users’ perspectives to guide the application of evidence and theory, with a focus on ensuring engagement. In addition to guiding qualitative investigations, the PBA promotes an approach that respects autonomy and attempts to ensure an empathic understanding of the user is clear throughout intervention content.

In-depth qualitative research conducted in the development of Internet interventions for mental health remains relatively novel. The qualitative research that has been conducted has primarily been nested within randomized controlled trials (RCTs) of Internet interventions for depression (for a review see [38]). Whilst this research is valuable for developing broad principles to explore more generally, conducting in-depth qualitative research as part of development processes enables users’ views and experiences to be rapidly incorporated directly into the resulting interventions.

### The Current Study

In this paper we aim to describe the development of the unguided Internet intervention “Healthy Paths Through Stress” (Short name: Healthy Paths), designed to support primary care patients in reducing emotional distress. Sub-threshold depressive symptoms are prevalent in primary care patients, with estimates as high as 45% [39]. Despite not meeting criteria for major depression, these symptoms lead to substantial functional disability, and can lead to the onset of major depressive disorder [39,40]. Patients experiencing this level of symptom burden may be suitable for unguided interventions. However, the content of full Internet CBT might seem less relevant to these patients if their symptoms are not being driven by established cognitive and behavioral patterns characteristic of diagnosed depressive disorder [21]. Our objectives were to (1) draw on a common factor model to select and modify established theory and evidence-based approaches; and (2) use the PBA to ensure the resulting unguided intervention has the greatest potential for both engagement and effectiveness.

## Methods

### Applying a Common Factor Model

The five common factors we aimed to ensure our intervention, Healthy Paths, included and/or promoted are as follows: (1) a functional explanation for a person’s symptoms, (2) an aligning coherent therapeutic rationale for improvement, (3) therapeutic techniques for ameliorating symptoms that align with the rationale, (4) use of techniques and skills in the person’s daily life, and (5) attribution of improvement to the person’s increased skillfulness and understanding.

#### Factors 1 and 2: Selecting a Functional Explanation for Symptoms and Coherent Rationale for Improvement

It is common for general practitioners (GPs) to point to difficulties in diagnosing psychological disorders in patients, as many present with social and/or environmental issues and what seem to be “problems of living” [41]. As such we wanted to draw on an overarching approach to the content of the intervention that incorporated social and environmental stressors into the explanations provided. Ricardo F Muñoz has developed an approach to preventing and treating depression that may be particularly relevant for distress in primary care. For 39 years Muñoz has practiced and taught at San Francisco General Hospital (SFGH), a public hospital that primarily serves a low-income, ethnically diverse, multilingual population. This led to the development of an approach with explicit focus on the formidable stressors faced daily by the populations SFGH serves [11]. Therapeutic manuals were written with an emphasis on what Muñoz has termed “The Healthy Management of Reality” [42], an approach that incorporates social learning theory [43], particularly reciprocal determinism and elements of CBT. The central tenet is that it is possible for individuals to use their thoughts and behaviors to manage their reality in a “healthy” way, that is, a way likely to lead to good outcomes for the individual and those around them [42]. There is an overt focus on individuals’ external reality (stressors), and internal reality (thoughts, feelings, and moods), ensuring rationales apply to those whose psychological symptoms may primarily be a response to crisis, acute or chronic environmental stressors such as illness, unemployment or familial issues, all of which are prominent in primary care practice presentations.

#### Factor 3: Therapeutic Techniques Aligning With Explanation and Rationale

Muñoz’s depression prevention manuals formed a basis for selecting techniques that we considered most suitable for unguided use. Suitability was based primarily on (1) the simplicity of the technique; (2) the likelihood of reinforcement during or soon after the practice; and (3) inclusion in evidence-based interventions. The first two factors are paramount, as patients are likely to be attempting to engage with material when their moods are low or they are experiencing emotional upset, often in the presence of enduring life stressors. Without the support and guidance of a therapist, techniques that are difficult and/or likely to be beneficial only after days or weeks of consistent use, may lead to disengagement and reduce effectiveness [5]. Based on these parameters cognitive restructuring techniques were excluded and self-monitoring techniques were included as potentially helpful, but made optional. Simple behavioral activation techniques were included as key strategies. Behavioral activation is an important part of Muñoz’s manuals [44], and is noted for both its simplicity and effectiveness in the treatment and prevention of depression [45]. A number of techniques from mindfulness-based approaches were also incorporated including body scanning, 3-minute breathing spaces, walking with awareness, and simple self-compassion exercises, all of which met our above criteria.

With the overarching rationale focusing on the healthy management of reality, we wanted to ensure the individual techniques were contextualized in a systematic framework, to strengthen coherence and avoid the tools provided appearing like a collection of disparate techniques. We proposed that helpful thoughts and behaviors fall into two groups: increasing awareness and making changes. Increasing awareness includes strategies such as self-monitoring (increasing awareness of thoughts and behaviors in the past), and mindfulness-based approaches (increasing present moment awareness of thoughts and behaviors). Making changes includes behavioral activation strategies (increasing the frequency of pleasant activities), simple cognitive tasks (increasing the frequency of helpful thoughts), and self-kindness practices. We suggested that both approaches worked well together, but that people may be drawn to one over the other. Emphasis was placed on encouraging the use of approaches individuals found engaging and useful.

The evidence to date suggests that in linear unguided programs, most individuals tend to use the first session, and then not go further [6,14]. This is an issue if the main therapeutic techniques in the program are introduced in later sessions. In addition, Donkin et al [46] found that there does not appear to be a simple linear relationship between total use or suggested techniques and the measurable benefit. This relationship is complex and still not sufficiently understood. To acknowledge this, we suggested our intervention should be used when it was needed, and all therapeutic techniques could be accessed immediately. The only requirement we made was that individuals should be tunneled through the introduction and rationale sections of the intervention on their first login, a requirement based on the importance placed on the therapeutic rationale by common factors models [25,28]. The introduction and rationale sections could be skipped at subsequent logins. Linear ways of using the techniques were suggested for those who might value this approach, but primarily the emphasis was on using the intervention in a way that worked best for the individual. This is consistent with an autonomy-supportive approach [47], which is likely to be critical for promoting engagement with unguided programs. In addition, there is evidence that Internet intervention users are able to pick the elements (kernels) within a participant preference trial that are relevant to their needs, and that when they do so, they are more likely to reach their goal [48].

In order to have some variance in a system that is non-linear, following the tunneling through the rationales, 3 routes or “paths” were provided to a range of specific techniques grouped under increasing awareness or making changes. Path 1 enables users to explore all the techniques and choose for themselves. Path 2 provides examples of different ways of dealing with difficulties, and recommends increasing awareness or making changes, depending on which way of dealing users feel applies most to them, whereas path 3 provides an emotion-specific recommendation option; users select their dominant feeling at the time (eg, sadness, anger or worry), and the intervention makes a recommendation based on that selection.

Healthy Paths could be described as having a modular structure, as it fulfills many of the criteria Chorpita et al [49] require for modularity. Healthy Paths consists of 12 “content modules” containing a therapeutic technique that could be used alone, or in combination with others, and 4 coordination modules (the introductory module and 3 paths modules) that provide overarching ways of thinking about the Healthy Paths approach, and guidance on which content module to select. A key difference with the examples of modular protocols described by Chorpita et al [49] is that in Healthy Paths the user self-guides regarding the choice of modules, rather than a therapist who assesses patients’ needs. There is also overlap with our focus on techniques and Embry and Biglan’s [50] concept of selecting evidence-based “kernels”; irreducible components that may affect symptoms or behavior change.

We aimed to make going through the intervention a visually reinforcing and appealing experience to enhance intrinsic motivation to use the material. Beyond text and audio content, we included hi-resolution nature images on the majority of the pages. As well as adding to the general positive feel of the intervention, there is a body of evidence to suggest that viewing nature images facilitates cognitive restoration [51], and can also improve mood [52]. The rationale for the images was explained in the intervention.

#### Factors 4 and 5: Use of Techniques and Skills in the Person’s Daily Life, and Attribution of Improvement to the Person’s Increased Skillfulness and Understanding

These important common factors are inherent in unguided Internet interventions. In face-to-face psychotherapy, improvement may be attributed to the therapist, whereas in antidepressant treatment, improvement is often attributed to the medication. When the primary delivery mechanism is text and audio, there is great potential for users to attribute improvements to their own development and new techniques learned.

### Intervention Logic Model

**Figure 1:**
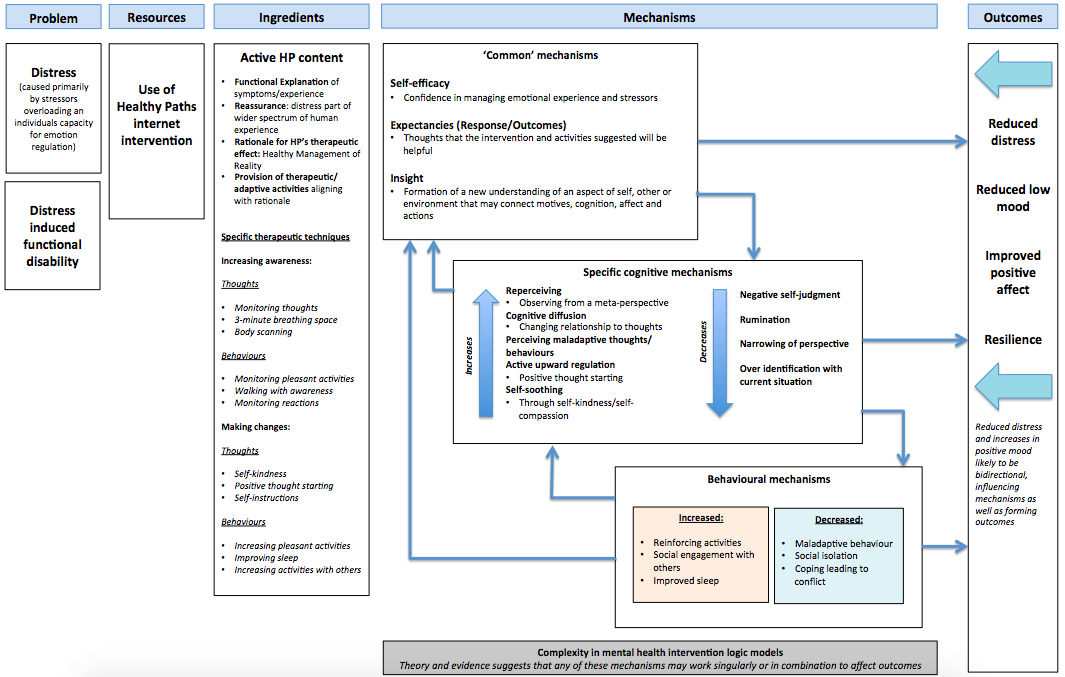
A logic model for Healthy Paths.

Creating logic models as part of the development process highlight assumptions’ researchers make about their interventions and how they produce change in symptoms. Logic models developed in public health interventions are now explicitly recommended in UK guidance on process studies [53]. Essentially, they are a visual representation of how interventions and their components may affect change (ie, the proposed mechanisms). We suggest that explicitly outlining logic models early in the intervention development cycle may facilitate discussion and debate regarding critical ingredients, and how interventions might be amended to become more suitable for different contexts, based on proposed mechanisms. A logic model for Healthy Paths can be found in Figure 1.

### Using the Person-Based Approach

The PBA has a number of elements that can be applied to intervention development, systematizing the involvement of target user groups. The three that will be discussed here are (1) exploratory qualitative interviews; (2) guiding principles; and (3) think-aloud interviews with early intervention prototypes. All elements are iterative, often occurring in parallel. Guiding principles are developed and amended throughout the process. Importantly, our aim is to use the PBA to inform the application of theory and evidence-based techniques in our unguided Internet intervention. This emphasis differs from how some may use participatory or co-design methods, with users themselves recommending and generating content.

#### Exploratory Qualitative Interviews

We conducted in-depth qualitative interviews to explore primary care patients’ experience of distress and to ensure our theory and evidence-based material was contextualized appropriately. Full methodological details have been described elsewhere [54]. Briefly, 20 patients were recruited from 10 UK primary care practices. Practices were asked to identify patients who had consulted in the last 12 months and the GP had placed a “distress” code in their notes, but did not diagnose with major depressive disorder or treated with antidepressants. Patients with a Patient Health Questionnaire-9 (PHQ-9) score of greater than 14 were excluded on the basis of likelihood of major depressive disorder. Interested patients consented to participate and were interviewed at their home or at their primary care practice. The first part of the interview was exploratory and focused on their experience of distress more generally (for full details see [54]), as well as their perceptions of Internet interventions. Interviews were conducted iteratively, in blocks of 3 or 4 over 2 weeks across a 9-month period. The interviews ranged in length from approximately 1 to 1.5 hours. A semi-structured interview guide was used and amended as the interviews went on to focus on points of interest. Patients provided informed consent, and the study was approved by the National Health Service (NHS) Research Ethics Committee (REC reference: 12/SC/0352). All interviews were fully transcribed, and a thematic analysis [55] was conducted with the resulting interview data.

#### Think-Aloud Qualitative Interviews

From the above sample, 13 patients undertook qualitative think-aloud interview [56]. In this interview participants went through early prototype versions of Healthy Paths on a laptop, with a researcher (JMS) who prompted them to “think-aloud” as they went through each page. In addition to assessing the usability of the intervention, participants were encouraged to describe their perceptions’ of the rationales, therapeutic techniques, and how these related to their experience of distress. All think-aloud interviews were transcribed verbatim.

For the current paper, the primary analyses included data from the open-ended interviews regarding participants’ views on Internet interventions in general and all think-aloud data. A thematic analytic approach was taken [55], drawing on methods of constant comparison [57]. AG read and reread all transcripts, and proceeded with open coding. A coding manual was developed by AG and reviewed by JMS. All potential amendments to the coding manual and themes were discussed between AG and JMS and added where there was agreement.

## Results

For a description of the participants’ baseline characteristics, see Table 1.

**Table 1:** Demographic characteristics (N=20).

Demographic characteristic		Participants, n (%)
Age in years, mean (SD)		51 (16.5)
Gender, female		15 (75%)
**Marital status**		
	Single	4 (20%)
	Married	10 (50%)
	Cohabiting	1 (5%)
	Divorced	4 (20%)
	Widowed	1 (5%)
**Ethnicity**		
	White	18 (90%)
	Indian	1 (5%)
	Other	1 (5%)
Age since left education, mean (SD)		18 (2.9)
**Qualifications**		
	No formal educational qualifications	3 (15%)
	GCSE^a^/O levels or similar	3 (15%)
	A levels or similar, ONC^b^/OND^c^	2 (10%)
	Diploma (non degree) HNC^d^/HND^e^	3 (15%)
	Degree	5 (25%)
	Postgraduate degree	1 (5%)
	Other	3 (15%)
**Employment**		
	Full-time employment	7 (35%)
	Not in employment due to long-term sickness	2 (10%)
	Part-time employment	4 (20%)
	Retired	4 (20%)
	Self-employed (full-time or part-time)	2 (10%)
	Unemployed	1 (5%)
**Income, £**		
	<10,000	3 (15%)
	10,000-20,000	3 (15%)
	20,001-40,000	8 (40%)
	>40,000	5 (25%)
	Not provided	1 (5%)

^a^GCSE: General Certificate of Secondary Education.

^b^ONC: Ordinary National Certificate.

^c^OND: Ordinary National Diploma.

^d^HNC: Higher National Certificate.

^e^HND: Higher National Diploma.

### Exploratory Interviews

The exploratory interview findings on participants’ experiences of distress are described in full elsewhere [54]. In summary, participants reported severe affective experiences, despite not being diagnosed with depression. The majority of patients’ disruptive affective experience was tied to complex life difficulties including chronic illness, caring roles following family or spouse serious mental and/or physical illness, unemployment, bereavement, bullying at work or relationship breakdown. Often multiple stressors coincided leading to the consultation with the GP. These stressors were described as overwhelming participants’ ability to cope. Terms like “depression” were used with different meanings (mood vs mental illness); however, a number of participants were cautious and sought to distance themselves from notions of “depression.”

Based on the exploratory interview data on patients’ experience of distress, the tone of the material was adjusted to ensure it was positive but acknowledged the difficulty and/or seriousness of stressful life events likely to lead to use of the interventions. The use of cartoons was avoided throughout, on the basis that they could be perceived as making light of users’ circumstances. Descriptions of difficulty were grounded in life experiences, and we avoided the use of words like depression and mentions of mental disorder or illness.

Analysis of patients’ perceptions of Internet interventions in the exploratory interviews resulted in 4 primary themes, which are discussed in turn below.

#### Positive Perceptions

The majority of participants were positive about the idea of using Internet interventions to manage distress, suggesting that Internet interventions could be used as a source of guidance and coping strategies in times of difficulty.

So it is having that sort of guidance as well and I mean even now I write stuff down so that I can go back to that. So if you have got it on the Internet you can go to it and, you know, give yourself...get yourself back.

Female, 26

I think a lot of problems is just having a lack of knowledge or information......or it could be like having coping strategies and things like that. Um, so if those could be fulfilled then yeah, I can’t see there being a problem.

Male, 40

#### Managing Flare-Ups

Some participants suggested that they would use an Internet intervention as a resource to manage “flare-ups” in their affective experiences. This supported the idea of proposing Healthy Paths be used as and when patients feel is necessary. It also provides a possible indication of why linear, unguided multisession interventions may be used infrequently by those with sub-threshold symptoms.

I mean at the moment, yeah, I would look at it um and if I did have another flare up, yeah, I probably most certainly would have another good look at it but um, I don’t think it will be something that I would continually... because I tend to when it’s gone, that’s it forget it and just carry on with things.

Female, 61

It’s there to sort of when, you know, when the alarm is going off I should go ok, just to get some perspective on what’s happening and just to quiet the alarm down for a minute... what would be key is to have in people’s minds is this trigger, stress um, go to that Internet site and then the sooner that can happen the sooner they can work through whatever is stressing them out.

Male, 39

#### Concerns

There were a small number participants who had concerns about Internet interventions. These concerns centered on self-help websites replacing time with a GP, and the necessity for face-to-face contact when dealing with emotional problems. When recommending Healthy Paths, it would be important for GPs to ensure patients understand that the intervention is in addition to, rather than in place of their usual primary care.

I think it’s possibly just a way of um taking away face-to-face contact with people.

Female, 50

#### Trust and Credible Source

Some participants talked about the importance of trusting the source of the information, when considering whether they would use an Internet intervention to manage distress.

That is something that I would… because I’ve spoken to you, yes, I would go on there because I know it is genuine… and it has come from the doctor as well, so it does make it totally different.

Female, 63

As Healthy Paths has been developed initially for primary care patients, although delivered as unguided, it is likely to be recommended by a GP. This link to a trusted health care practitioner via a trusted health care pathway may increase uptake and engagement.

### Development of Guiding Principles

On the basis of our exploratory qualitative interviews, theory, and evidence a set of guiding principles were developed for Healthy Paths (Textbox 1). Guiding principles, features of the PBA, are short statements of intervention design objectives and key features of the intervention design that can meet these objectives. The aim of guiding principles is to succinctly capture what is unique or distinctive about the intervention. As such, they are useful when working on development in large multidisciplinary teams, particularly where development will occur in iterations over a number of years; ensuring members stay on track and work toward the same goals.

**Guiding principles for the Healthy Paths intervention.**

Intervention design objectives and the key features that may achieve these objectives

- To design material for an unguided context, focusing on simplicity and maximizing intrinsic motivation to engage.
  - Ensure material is simple, informative, and as original as possible.
  - Selected techniques should have potential for reinforcement or to be rewarding with little behavioral commitment.
  - Navigation should be straightforward, whilst providing different ways of engaging with the material.
  - Autonomous motivation will be fostered by:
    - Providing choice;
    - Giving explanations for why suggestions might be helpful;
    - Using non-directive language throughout such as a tone that invites rather than instructs [35].
- To offer material suitable for broad range of primary care patients experiencing emotional distress.
  - Include content with a primary focus on core feelings, thoughts, and behaviors common to many distress experiences, with an acknowledgment of environmental and social causes.
  - Included content will seek to demedicalize experiences, through avoidance of "terms such as “mental illness” and “psychological disorder.”

### Think-Aloud Interviews

Analysis of think-aloud data resulted in 6 positive themes relating to aspects of the intervention and 4 negative themes relating to difficulties. The negative themes guided intervention amendments. Primary themes with examples are summarized below.

#### Look and Feel

Throughout the think-aloud interviews, participants were generally positive about the look and feel of the intervention. In particular participants often suggested the look and layout was calming.

I like the whole look of it actually because it feels calm. It sounds stupid but the colors are right.

Female, 61

Given that it's to do with stress it seems quite nice and calming; yes, nice layout, easy to read so the layout is nice.

Female, 39

#### Specific Intervention Explanations and Rationales

The majority of participants suggested that the therapeutic rationales were clear, and the ideas made sense. Participants talked about the importance of having different options to support them through their experiences. They also mentioned the importance of simplicity and messages being to the point.

I think that's a good idea because it is different for different people and it is a path and it takes a long time and, yes, I think that's a good way of putting it, actually. It's not an immediate thing and a path, it's a way to go; it might be a short time, it might be a long time and that is the best way of describing it, really.

Female, 26

It's nice and quite to the point, there's not too many hidden meanings or anything behind what the message is, so that's good.

Female, 24

#### Content in General

Participants reported feeling that they could relate to the content. They valued the direction the intervention provided and emphasized the positive aspects of having the information broken into small sections.

Right, you see that’s quite good, I think, isn’t it really, because it’s saying what are you like, right ok, this is the path for you. You know, that’s directing you. I think that’s quite good.

Male, 68

If I came in and said, “Well, I don’t know what to do”, supposing it happened to me, I’d think well, what should I do? And then I’ve had a positive feedback to tell me to do such and such a thing...

Female, 88

I think that the information is interesting. It's broken into chunks, easily absorbed, especially I'm just thinking if I'm feeling more stressed than I am now then it is nice and calming to read it and it's inviting to read it.

Female, 39

#### Usability Difficulties

On early versions of the intervention, some participants suggested that rationales were too extensive, and they were looking for a way to get directly to techniques to help them. Subsequently, the intervention was amended to signpost how the intervention would work, and inform users that following the first login, they could skip all rationales and go directly to the therapeutic techniques.

I think really people who are anxious or worried they’re not thinking straight at that time are they, their thoughts are irrational, so are they going to sit down and plot through something like this when all they want is relief from the way they are feeling? So I think a straight line to the techniques—yeah, I agree you have got to put all of the background in and the understanding and why it is happening and all that, and that’s going to be beneficial to a lot of people—um, but I think, you know, cutting to the techniques...

Male, 66

#### Concerns With Images

Initially, a small number of nature images were used to illustrate feelings that were being discussed in the content, for instance dark clouds for low mood. Some participants did not like these images, leading to their removal. We then ensured all nature images depicted uplifting scenes.

Dark and dismal. I don't like it. [what about] a nice blue sky, it's sky isn't it, and clouds? A bluer sky, nicer, I'd rather see. Maybe I'm wrong, maybe when you're – no that would make you even lower.

Female, 69

#### Complexity of Text

In the increasing awareness section (based on mindfulness approaches), some participants suggested that rationales were too complex. Following this being noted, all sections were broken down to ensure the material as was straightforward as possible.

[P reading material] “The difference with walking with awareness is that we deliberately chose to notice more of the actual experience of walking” Yeah. I just think that that could be said in a more simplistic way. And some people may appreciate that.

Male, 66

#### Issues With Explanations

Although all efforts were made to ensure simplicity in the description of ideas, if the idea itself was very straightforward or not novel, it was picked up as being “obvious.”

It’s stating the obvious ain’t it, ‘Helpful thoughts help you achieve a positive mood, harmful thoughts produce a more negative mood’.

Male, 66

As such, efforts were made to reduce or remove these obvious statements throughout the intervention. Where the team felt it was important to keep them, we ensured this was acknowledged, for instance, by prefixing a section with “this may seem obvious…” and going on to explain why it was still important to mention.

**Figure 2:**
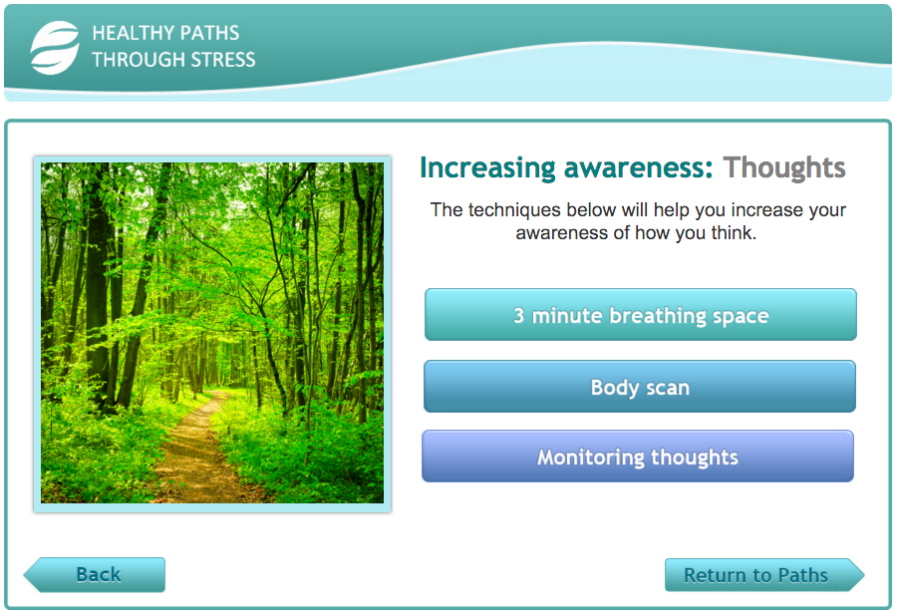
Screenshot of Healthy Paths.

Overall, both the open-ended and think-aloud interviews were used to ensure the theory and evidence informed intervention was thoroughly grounded in the lived experiences of the target users. A screenshot from the intervention is shown in Figure 2.

## Discussion

### Principal Findings

This paper describes our approach to developing an unguided Internet intervention. Common factor meta-theory guided the selection of and amendments to the evidence-based content, with the aim of retaining effectiveness in an unguided context. The PBA was used to guide the integration of users’ perspectives into the development process, as well as ensure material fostered autonomy. Both exploratory and think-aloud elements led to amendments and adaptations to the theory- and evidence-based content aimed at improving engagement. Subsequently, the majority of participants found our intervention to be informative, able to relate to their experiences, and to be acceptable.

In Healthy Paths, users are encouraged to engage with the intervention in a way that suits them, particularly in times of need. This deviates from approaches employed by previous researchers, where Internet interventions for mental health are structured so that sessions or modules recap and build on each other in a progression over time [58,59]. Our aim is to foster intrinsic and autonomous motivation to use the intervention as far as possible. Avoiding the learning course structure may be important to reach and retain those with lower levels of educational attainment, where elements reflective of a more extrinsic approach (eg, completing sessions within set time intervals, homework worksheets etc) may be particularly off-putting. If initial interest is driven by a user’s autonomous motivation to reduce their symptoms, and then when using the intervention they find it to be a positive, reinforcing experience, this may lead to sustained motivation for use in the future. This approach acknowledges that symptoms are often a primary driver of adherence and engagement across a number of areas; the presence of pain is related to greater adherence to physiotherapy exercises [60], and increase in symptoms is linked to greater adherence to antidepressant medication [61].

We believe this is the first application of the PBA to the development of an Internet intervention targeting mental health. Our approach to development differs substantially from other development examples. For example, Landback et al [62] used a product design methodology without qualitative user input when developing an Internet intervention for preventing adolescent depression in primary care; Sheeber et al [58] developed and modified their intervention for preventing post-partum depression primarily on theoretical grounds; and Tiburcio et al [63] developed an intervention for substance abuse and depressive symptoms including 20 users, although their input did not go beyond usability aspects of the intervention. The PBA ensures sound usability testing principles are enriched with users’ experience of their symptoms, and their perceptions regarding the suitability of approaches in relation to those symptoms.

The development of a logic model [53,64] for our intervention represents a novel application of this approach to the mental health field. With developer’s assumptions clear from the outset, it becomes easier to observe and address challenges, and subsequently adapt theory collaboratively. In combination with in-depth user perspective, this theory and person-based approach may take us closer to understanding why Internet interventions produce change, and how they can be improved on this basis.

The initial development of Healthy Paths represents the first step in a continuing, iterative research program. Future projects include a longitudinal qualitative study of primary care patients experiencing distress using Healthy Paths over a 4-week period. Patients will be interviewed over this month regarding how they are using the Internet intervention and the suggested techniques. After this there will be another round of optimization, in preparation for large-scale primary care trials. If successful, Healthy Paths may be suitable for the first step of stepped care programs [65], and will also be openly disseminated free of charge as an example of a massive open online intervention (MOOI) [12]. With a primary focus on managing difficult emotions, Healthy Paths may also be easily adapted for specific stressful circumstances (for instance, managing illness-related distress).

### Limitations

Our work has some limitations. The sample used in the qualitative work was predominately female. In future work we will focus on sampling equal numbers of males. In addition, we are likely to have recruited patients who are disposed to this form of intervention and delivery. In the next phase of this project we aim to develop recruitment materials such that we reach beyond this group, in order to include a more diverse range of perspectives. There were a smaller number of participants who took part in think-aloud interviews, potentially limiting the perspectives provided. As common factors models operate at a meta-theoretical level they are necessarily broad, increasing difficulty in application. This puts the onus on researchers to provide and extrapolate their therapeutic rationales for others to evaluate and judge whether they are to be considered “coherent,” for instance.

### Conclusion

Unguided Internet interventions have great potential for improving mental health if they can be developed to be engaging and effective. The development of Healthy Paths represents the application of novel approaches, encouraging debate around intervention development and mechanisms of effectiveness. Ultimately, we hope our work will lead to new a wave of effective unguided self-directed therapeutic resources for highly prevalent psychological distress.

## Abbreviations

CBT: cognitive behavior therapy
EBP: evidence-based psychotherapy
e-mental health: electronic mental health
GP: general practitioner
NIHR: National Institute of Health Research
PBA: person-based approach
SFGH: San Francisco General Hospital
